# Report of an Experiment With a Fetal Ex-Utero Support System in Piglets

**DOI:** 10.7759/cureus.38223

**Published:** 2023-04-27

**Authors:** Ayssa T Abrao Trad, Randal Buddington, Elizabeth Enninga, Jose Duncan, Claudio V Schenone, Giancarlo Mari, Karyl Buddington, Mauro Schenone

**Affiliations:** 1 Obstetrics and Gynecology/Maternal-Fetal Medicine, Mayo Clinic Alix School of Medicine, Rochester, USA; 2 College of Health Sciences, University of Memphis, Memphis, USA; 3 Obstetrics and Gynecology, Mayo Clinic Alix School of Medicine, Rochester, USA; 4 Obstetrics and Gynecology/Maternal-Fetal Medicine, The University of Tennessee Health Science Center, Memphis, USA; 5 Obstetrics and Gynecology, The University of Tennessee Health Science Center, Memphis, USA

**Keywords:** fetus, prematurity, ex-vivo support, artificial uterus, artificial placenta

## Abstract

Extreme prematurity remains one of the leading causes of neonatal death. An ex-utero treatment strategy that allows the fetus to develop beyond this period until capable of tolerating the transition to post-natal physiology would significantly impact the quality of care offered for this pre-viable patient population. In this study, we report our experience with an ex-utero support system for fetal pigs with the goal of support and survival for eight hours. Our experiment included two pigs at a gestational age equivalent to a 32-week human fetus. Following ultrasound assessment and delivery via hysterotomy, the fetuses were transferred to a 40 L glass aquarium filled with warmed lactated Ringer's solution and connected to an arteriovenous (AV) circuit that included a centrifugal pump and a pediatric oxygenator. Fetus 1 was successfully cannulated and survived for seven hours (expected maximum duration of eight hours). Fetus 2 died shortly after hysterotomy, secondary to failure at the cannulation stage. Our results suggest that ex-utero support of the premature fetal pig is feasible, contributing to a scarce body of evidence. However, further studies are needed before effectively translating an artificial placenta system into the clinical arena.

## Introduction

Prematurity, defined as delivery before 37 weeks gestation, is the second leading cause of death in children under five years of age and the single most important direct cause of death in the first month of life [1].

Overall outcomes for preterm infants have markedly improved over the past decades due to advances in neonatal and obstetrical care, including antenatal steroid therapy and postnatal administration of exogenous surfactant [2]. However, outcomes are still closely linked to gestational age at delivery, with survival to one year ranging from 6% at 22 weeks to 94% at 28 weeks, and significant morbidity occurring in 86% of the few survivors born at 22 weeks improving to 54% at 28 weeks gestation [3]. Fetuses delivered before 22 weeks are currently considered not viable ex-utero. Advances have been achieved toward the survival of extremely preterm infants (delivery before 28 weeks gestation). Still, critical unresolved issues and complications exist in managing infants born between 22 and 28 weeks, mainly due to lung immaturity and respiratory failure. During the mid-canalicular development phase (approximately 16-26 weeks gestation), the fetal lungs cannot effectively take charge of the gas exchange required for the current standard of care [4]. Stoll et al. suggest that available science has reached the limits of what current technologies can provide to compensate for lung immaturity for this specific patient population [2]. Novel concepts and areas of study are needed to address the challenges posed by extreme immaturity.

A treatment strategy designed to maintain fetal physiology outside the uterus and allow the premature fetus to develop until capable of tolerating the transition to postnatal physiology would significantly impact the quality of care for this patient population. Ideally, the fetal circulation and gas exchange would be preserved using a system that simulates the placenta and uterus, with arterio-venous (AV) extracorporeal life support connected to the fetus via the umbilical vasculature. Whereas previous efforts using fetal lambs have validated the concept of fetal incubation [5], pigs provide the advantages of greater translational relevance [6]. The large litter sizes allow for replication and comparisons of different strategies using littermates. We report the initial study of an ex-utero support system for fetal pigs with the goal of support and survival for eight hours.

## Technical report

Experimental protocol

This experiment included two pigs at 102 days gestational age (term 115 ± 1 day) [7]. At this gestational age, fetal pigs are comparable to the preterm human fetus of roughly 32 weeks gestation [8]. The production pigs used for this study have an average litter size of 13-14, and it is not rare for sows to have more than 20 fetuses in a litter. The sows are of a consistent genetic lineage, specific pathogen-free, and bred by artificial insemination, providing a known gestational age. Body weights of the fetuses are larger than those of minipigs (900-1,000 g vs. 300-400 g) but are markedly smaller than fetal lambs (3-4 kg) at the same stage of fetal development and are therefore more comparable to extremely or pre-viable preterm infants. The fetuses were assessed in utero by ultrasound to verify viability before beginning the procedure to deliver via hysterotomy. This study was completed in February 2017 after approval by the Institutional Animal Care and Use Committee of the University of Memphis. The study was conducted at the College of Health Sciences at the University of Memphis in Memphis, Tennessee, United States.

System components

**Figure 1:**
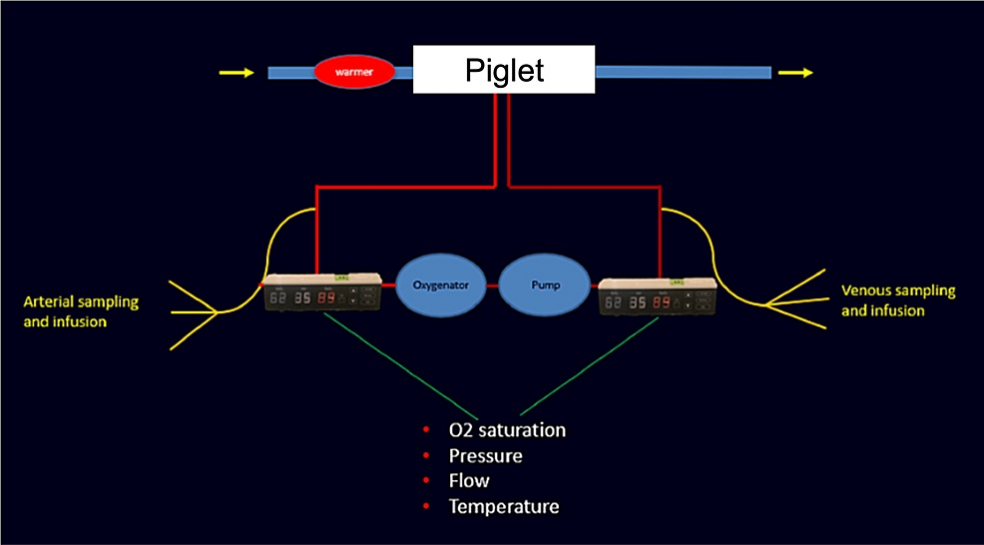
Schematic representation of the fetal ex utero support system using a warmer and AV circuit containing a centrifugal pump and a Pixie™ pediatric oxygenator AV: arteriovenous Pixie™ pediatric oxygenator, Medtronic PLC, Dublin, Ireland

**Figure 2:**
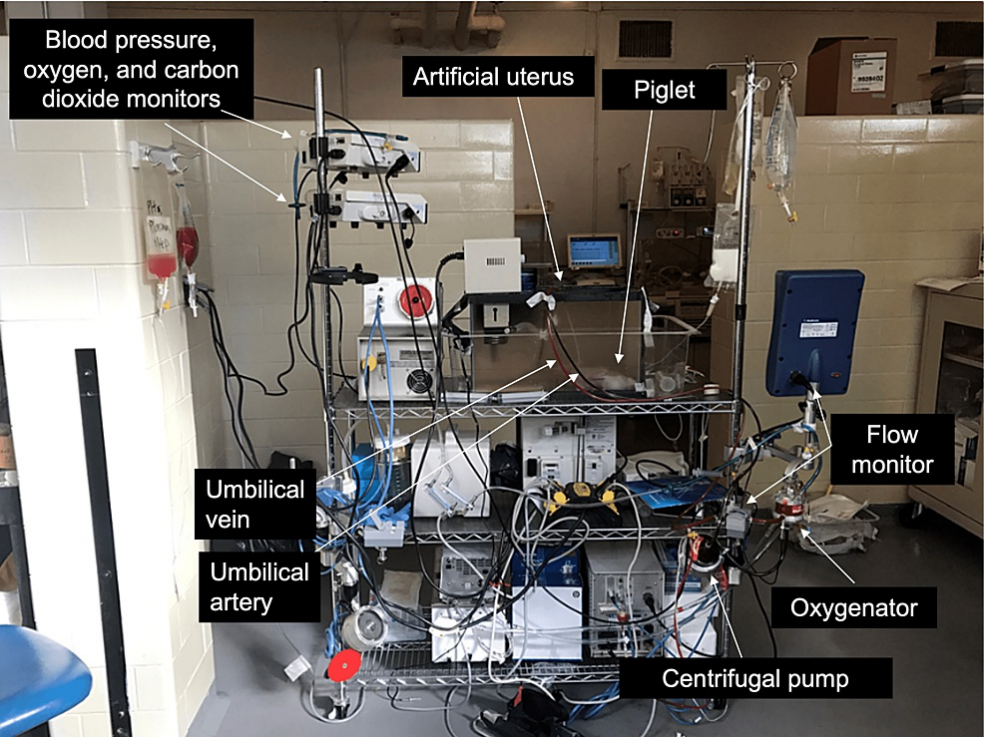
Fetal ex utero support system components

A 40 L glass aquarium with an open top was used as an artificial uterus. Lactated Ringer’s solution with 5 mmol glucose was used as a substitute for amniotic fluid and was warmed to 38-39 C using a recirculating heater. The fetal pigs were connected to an AV circuit consisting of a centrifugal pump and a Pixie™ pediatric oxygenator (Medtronic PLC, Dublin, Ireland) with an 82 mL priming volume. Heparinized blood obtained from the sow was used to prime the entire system with 200 mL. Continuous heparin infusion was not needed after the fetus was connected to the system, based on clotting times. Figure 1 and Figure 2 depict the details of the system.

Cannulation procedure and transition to the system

After a fetal pig was isolated while undelivered, a free loop of the umbilical cord was exteriorized through a small incision in the uterus. The fetal cord insertion into the fetus was determined to establish the direction of catheterization. While the fetus was still on placental support, the surgeons skeletonized the umbilical vessels and subsequently made a nick incision in one of the two umbilical arteries while clamping off the cord. A vein pick catheter introducer facilitated the introduction of the umbilical vessel catheter (6.5 Fr in fetus 1 and 8 Fr in fetus 2; Umbili-Cath™ (Utah Medical Products Inc.)) to a depth of about 10-12 cm. The catheter was secured using sutures and connected to the inflow port of the primed oxygenator before a similar approach was used to place and secure a catheter in the umbilical vein (8 Fr; Umbili-Cath) and connect it to the outflow port of the oxygenator. The centrifugal pump was turned on, and flow was confirmed. The second umbilical artery was then catheterized, secured, and connected to the inflow port. The reasoning behind attempting an additional catheter included possible use for parenteral nutrition and invasive blood pressure monitoring or to parallelize the system, if necessary, for adequate flow. The fetal-placental circulation was then terminated, the cord was cut, and the fetus was rapidly transferred to and submerged in the artificial uterus without gasping. 

Support and monitoring

Fetuses were evaluated for appearance and movement. Samples of umbilical artery blood were obtained to analyze fetal blood gases and chemistries. Fetal blood flow was assessed continuously by a flow meter connected to the Bio-Console™ 560 Speed Controller System (Medtronic PLC). Saturation and hematocrit were also assessed continuously by the BioTrend® Oxygenation Saturation and Hematocrit Monitor (Medtronics PLC). Necropsy was performed after fetal death confirmation.

Results

Fetus 1

Catheterization of the first umbilical artery and the vein was successful, with the cord accessed at approximately 6 cm from insertion into the body. The attempt to catheterize the second umbilical artery was not considered successful based on the lack of blood flow into the catheter. The functioning arterial and venous catheters were connected to the system, and the fetus was submerged in the artificial amniotic fluid. 

The system successfully maintained adequate activated clotting time during the entire experiment without clotting (seven hours). The fetus had excellent color (pink, suggestive of good peripheral tissue oxygenation) for at least seven hours, moved spontaneously, and responded to touch. The umbilical blood flow remained at 80-90 cc/min, within the lower limit expected for a 1-1.2 kg fetal weight. Due to issues with perfusion and hypovolemia, three boluses of lactated Ringers of 10-20 cc were administered at the second, fourth, and sixth hours; this caused transient increases in flow. Lactate levels rose to > 15 mmol/L after the second hour of the experiment and remained above the detection limit for the remainder of the incubation. We administered two doses of bicarbonate to address decreasing blood pH; Table 1 depicts pH and bicarbonate analysis throughout the investigation.

**Table 1:** pH and bicarbonate analysis in Fetus 1 after transference to the system

Time	pH	Bicarbonate (mmol/L)
12:50	7.4	13.7
13:43	7.51	>15
HCO_3_ administration		
14:20	7.68	>15
15:03	7.53	>15
16:51	7.25	>15
18:08	7.22	>15
HCO_3_ administration		
18:58	7.09	>15
19:21	6.94	>15

At around seven hours and 20 minutes of fetal support, we noted a sudden loss of flow through the system, and the fetus was unresponsive to resuscitative maneuvers, such as repositioning of the umbilical cord and catheters to ensure no kinking and a bolus of normal saline. Shortly thereafter, fetal death was confirmed. Although the cause of death was not determined, excessive lactate was suspected based on the decline in blood pH.

A gross examination was performed on necropsy with unremarkable results. The umbilical artery catheter was noted in place (within the intrafetal portion of the umbilical artery). Similarly, the umbilical vein catheter remained in place with the tip at the level of the fetal abdominal wall. As expected, the ductus arteriosus was patent, and the lungs were consolidated.

Fetus 2

We exposed the umbilical cord and followed the same approach for cannulation as Fetus 1 but used larger (8 Fr) umbilical artery catheters. Blood flow in the two umbilical artery catheters was observed before connection to the circuit. Despite that, flow in the circuit was not established, even with the pump activated. The fetus was transferred to the incubation tank, but without any established flow, and death was ascertained soon after. Necropsy was not performed. Dislodgment of the catheters was the likely cause of death.

## Discussion

Despite the many advances in prenatal and postnatal care, morbidity and mortality of extremely premature newborns remain very high [3]. This is attributed to the associated organ immaturity not being compatible with ex-utero life. An artificial womb and placenta that prolongs fetal growth and organ maturation and delays the transition to postnatal physiology will significantly reduce morbidity and mortality. We report an initial experiment using preterm pigs as a translational model to evaluate if an artificial placenta and uterus system will maintain fetal physiology ex-utero.

Historical perspective

The concept of an artificial placenta is not new. Initial reports date back to the 1950s and 1960s [9], with many attempts and system modifications proposed over time [9-16]. Callaghan et al. reported a pioneering effort in ex-utero fetal support in 1961 using a rotating 5-inch disc oxygenator (Pemeo), Davol pump, and a reservoir [10]. In 1965, the same group reported the first pumpless system. This approach supported the fetus for less than three hours [17], and the discouraging results led to a reliance on systems with extracorporeal pumps. Zapol et al., in 1969, extended the length of survival on ex-utero fetal support to two days, using a heparinized system (priming volume of 240 cc) with a roller pump, membrane oxygenator, umbilical cannulation, controlled temperature, bath of synthetic amniotic fluid, antibiotics, and nutrients (infusion glucose, amino acids, and vitamins) [9]. More than a decade passed before more reports of fetal incubations appeared in the late 1980s. In 1987, Kuwabara et al. reported the first seven-day survival of a fetal lamb on an ex-utero fetal support [5]. The same group, in 1989, achieved survival of 9.8 days after modifications that included a dialysis system and deeper insertion of the arterial catheter into the dorsal aorta [11]. This was followed by achieving three-week survival in 1997 [12]. After another relatively silent decade in the ex-utero fetal support literature, in 2009, Reoma et al. reported limited survival of four hours using a pumpless system with flow probes placed in the aorta and ductus arteriosus [14]. Miura et al. extended survival to 29 hours with a pumpless system [18]. Survival was extended further to 70 hours by Gray et al. in 2013 using a dry bed system, intubating the fetus, cannulating the jugular vein and umbilical vein (venous-venous extracorporeal life support), and ligating the umbilical arteries [15]. Partridge et al. used a pumpless system and hollow fiber oxygenator incorporated in a closed circuit with total parenteral nutrition to extend survival for up to four weeks with stable hemodynamics, normal blood gases, adequate oxygenation parameters, and patency of the fetal circulation. With appropriate nutritional support, fetal lambs connected to this system demonstrate physiologic somatic growth, lung maturation, brain growth, and myelination [19]. More recently, system adjustments have allowed support of ovine fetuses delivered earlier in gestation, with lung development equivalent to a 24-week-old human fetus [16].

Despite the successes in previous experiments, there are limitations to using fetal lambs as a model for extremely preterm human infants. Importantly, to address fetuses during the canalicular phase of lung development, the fetal lambs are studied between 94-110 days of gestational age, and their weight is approximately 1 kg, which is roughly twice the size of a human fetus in the same phase of development, whereas fetal pigs are comparable in size. Since the fetal size is associated with hemodynamic factors, this difference might significantly impact relevance and transitioning to clinical applications. Advantages of the fetal pig model include the fact that the vascular anatomy of fetal pigs is closely related to that of humans, with the same number of arteries and veins within the umbilical cord (in contrast, sheep have two umbilical arteries and two umbilical veins). From a practical perspective, fetal pigs are available throughout the year. Preterm pigs can extend the successes with fetal lambs, facilitate further advances, and enhance translation into clinical settings.

Our experience

Amongst the critical lessons learned from fetal lambs and our experiences with fetal pigs is that cannulation via the umbilical cord can be challenging, particularly when working within a limited time. Innovative approaches are needed to cannulate and secure the umbilical cord vessels more efficiently. The optimal depth of insertion of the tip of the umbilical arterial and venous catheters still needs to be determined. We advanced the venous catheter to the intraabdominal umbilical vein, not reaching the ductus venosus. Other teams have had significant success leaving the tip of the catheter a few centimeters outside the abdominal wall [19]. Imaging approaches, such as radiographs, may be used to confirm the positioning of the catheter tips. However, even when successfully cannulated, the fetus may move and cause dislodgement leading to fetal death. Sedation during periods of significant fetal agitation and securing mechanisms may be considered to avoid cannula dislodgement.

The pump may either limit or produce excessive flow and affect fetal heart function. A pumpless system relying on the fetal heart for blood flow is desirable. A system using the fetal heart as the pump must have low resistance and a low priming volume (<30cc), which is closer to the average fetal-placental volume. Low priming volumes will also minimize reliance on large volumes of blood from the sow (maternal) or other donors.

The optimal blood gases for the preterm infant remain unknown [20], with even less known for the fetus. To better understand the fetal pig, while delivering preterm pigs of the same gestational age, we measured blood gases in blood samples collected from the umbilical vein and one of the two umbilical arteries (using a blood gas analyzer, GEM® Premier™ 3500 (Werfen, S.A., Barcelona, Spain) before clamping the cord (Table 2).

**Table 2:** Blood gases for venous and arterial blood collected at 102 days gestational age, before cord clamping

Origin	pH	CO_2_ (mmHg) (mean ± SD)	O_2_ (mmHg) (mean ± SD)	Lactate (mmol) (mean ± SD)
Venous (from the placenta to the fetus)	7.2 ± 0.0	111.7 ± 2.1	63.0 ± 9.2	2.5 ± 0.2
Arterial (from the fetus to the placenta)	7.2 ± 0.0	>115	27.2 ± 6.0	2.3 ± 0.3

These values represent fetal values and provide insights into the transplacental gas exchange. Achieving oxygen saturation (SpO2) >65-85%, arterial oxygen pressure (PaO2) of 25- 5 mm Hg, and partial pressure of carbon dioxide (PCO2) of 35-45 mm Hg in the umbilical vein are considered desirable. An oxygenator with low resistance and a low priming volume that permits a gradual change in blood gases may enhance our ability to maintain stability. Fetal nutrition support is another area in need of research.

In our experience, using heparinized blood from the sow to prime the system was enough to keep the activated clotting time within the physiologic range for more than seven hours. This raises a question about the need for a continuous heparin infusion, especially in systems like ours with antithrombotic surfaces that increase the hemocompatibility of the circuit. We did not assess infection due to the short duration of the current experiment. We recognize that closed system designs are needed for prolonged incubations to avoid amniotic fluid and fetus contamination. Sealed bag systems with a single opening minimize the risk of microbial colonization [16] and facilitate the performance of ultrasound monitoring of the fetus (i.e., cardiac activity and fetal shunts). These are potential improvements that we are considering for our future experiments.

## Conclusions

We present a proof-of-concept of an ex-utero fetal support system using piglets. Our experience adds to the minimal literature on the artificial placenta using a pig model. We established that using tubing with anticoagulant coating decreases the need for heparin. Short-term hemodynamic stability is feasible using a pumped system. Additionally, we demonstrated the patency of fetal shunts upon autopsy. A multidisciplinary approach was critical to the success of our experiment. Medical disciplines such as maternal-fetal medicine, neonatology, cardiovascular perfusionist, and anesthesia are vital players. Further animal research is needed before this technology is ripe for human trials.

## References

[1] Liu L, Johnson HL, Cousens S (2012). Global, regional, and national causes of child mortality: an updated systematic analysis for 2010 with time trends since 2000. Lancet.

[2] Stoll BJ, Hansen NI, Bell EF (2015). Trends in care practices, morbidity, and mortality of extremely preterm neonates, 1993-2012. JAMA.

[3] Anderson JG, Baer RJ, Partridge JC (2016). Survival and major morbidity of extremely preterm infants: a population-based study. Pediatrics.

[4] Copland I, Post M (2004). Lung development and fetal lung growth. Paediatr Respir Rev.

[5] Kuwabara Y, Okai T, Imanishi Y (1987). Development of extrauterine fetal incubation system using extracorporeal membrane oxygenator. Artif Organs.

[6] Charest-Pekeski AJ, Sheta A, Taniguchi L (2021). Achieving sustained extrauterine life: challenges of an artificial placenta in fetal pigs as a model of the preterm human fetus. Physiol Rep.

[7] Cohen S, Mulder EJ, van Oord HA, Jonker FH, Parvizi N, van der Weijden GC, Taverne MA (2010). Fetal movements during late gestation in the pig: a longitudinal ultrasonographic study. Theriogenology.

[8] Eiby YA, Wright LL, Kalanjati VP (2013). A pig model of the preterm neonate: anthropometric and physiological characteristics. PLoS One.

[9] Zapol WM, Kolobow T, Pierce JE, Bowman RL (1969). Artificial placenta: two days of total extrauterine support of the isolated premature lamb fetus. Science.

[10] Callaghan JC, Angeles JD (1961). Long-term extracorporeal circulation in the development of an artificial placenta for respiratory distress of the newborn. Surg Forum.

[11] Kuwabara Y, Okai T, Kozuma S (1989). Artificial placenta: long-term extrauterine incubation of isolated goat fetuses. Artif Organs.

[12] Unno N, Baba K, Kozuma S, Nishina H, Okai T, Kuwabara Y, Taketani Y (1997). An evaluation of the system to control blood flow in maintaining goat fetuses on arterio-venous extracorporeal membrane oxygenation: a novel approach to the development of an artificial placenta. Artif Organs.

[13] Sakata M, Hisano K, Okada M, Yasufuku M (1998). A new artificial placenta with a centrifugal pump: long-term total extrauterine support of goat fetuses. J Thorac Cardiovasc Surg.

[14] Reoma JL, Rojas A, Kim AC (2009). Development of an artificial placenta I: pumpless arterio-venous extracorporeal life support in a neonatal sheep model. J Pediatr Surg.

[15] Gray BW, El-Sabbagh A, Zakem SJ (2013). Development of an artificial placenta V: 70 h veno-venous extracorporeal life support after ventilatory failure in premature lambs. J Pediatr Surg.

[16] Usuda H, Watanabe S, Saito M (2019). Successful use of an artificial placenta to support extremely preterm ovine fetuses at the border of viability. Am J Obstet Gynecol.

[17] Callaghan JC, Maynes EA, Hug HR (1965). Studies on lambs of the development of an artificial placenta. Review of nine long-term survivors of extracorporeal circulation maintained in a fluid medium. Can J Surg.

[18] Miura Y, Matsuda T, Funakubo A, Watanabe S, Kitanishi R, Saito M, Hanita T (2012). Novel modification of an artificial placenta: pumpless arteriovenous extracorporeal life support in a premature lamb model. Pediatr Res.

[19] Partridge EA, Davey MG, Hornick MA (2017). An extra-uterine system to physiologically support the extreme premature lamb. Nat Commun.

[20] Zoban P (2019). Optimal oxygen saturation in extremely premature neonates. Physiol Res.

